# Isolated ventricular septal defect is not a risk factor for celiac disease: evidence from a large real-world data cohort of 493,382 children

**DOI:** 10.3389/fped.2026.1751030

**Published:** 2026-03-18

**Authors:** Ramon Cohen, Haitham Abu Khadija, Shay Nemet, Mohammad Masu'd, Duha Najajra, Alena Kirzhner, Tal Schiller, Nizar Abu Hamdeh, Shira Bezalel-Rosenberg, Ilan Asher, Ali Abdallah, Keren Mahlab-Guri, Mohammad Alnees, Daniel Elbirt

**Affiliations:** 1 Department of Internal Medicine B, Kaplan Medical Center, Faculty of Medicine, Hebrew University of Jerusalem, Clalit Health Services, Rehovot, Israel; 2 Department of Clinical Immunology, Allergy and AIDS, Kaplan Medical Center, Faculty of Medicine, Hebrew University of Jerusalem, Clalit Health Services, Rehovot, Israel; 3 Department of Cardiology, Kaplan Medical Center, Faculty of Medicine, Hebrew University of Jerusalem, Clalit Health Services, Rehovot, Israel; 4 Department of Internal Medicine A, Kaplan Medical Center, Faculty of Medicine, Hebrew University of Jerusalem, Clalit Health Services, Rehovot, Israel; 5 Department of Diabetes, Endocrinology and Metabolism, Kaplan Medical Center, Faculty of Medicine, Hebrew University of Jerusalem, Clalit Health Services, Rehovot, Israel; 6 Department of Endocrinology, The Edith Wolfson Medical Center, Holon, Israel, affiliated with the Faculty of Medicine, Tel Aviv University, Tel Aviv, Israel; 7 Institute of Gastroenterology, Kaplan Medical Center, Faculty of Medicine, Hebrew University of Jerusalem, Clalit Health Services, Rehovot, Israel; 8 Harvard Medical School, Postgraduate Medical Education, Global Clinical Scholar Research Training Program, Boston, MA, United States

**Keywords:** autoimmune diseases, celiac disease, chromosomal abnormalities, congenital heart disease, epidemiology, pediatrics, type 1 diabetes mellitus, ventricular septal defect

## Abstract

**Background:**

Ventricular septal defect (VSD) is one of the most common congenital heart defects in children. Celiac disease (CD) is known to cluster with autoimmune conditions and chromosomal syndromes, but it remains unclear whether isolated VSD is independently associated with CD.

**Methods:**

We performed a population-based retrospective cohort study using electronic records from Clalit Health Services. Children aged 0–10 years were followed for up to 10 years to identify incident cases of CD. Multivariable Cox proportional hazards models were used to estimate adjusted hazard ratios (HRs) with 97.5% confidence intervals (CIs) for the association between VSD and CD. The models were adjusted for age, sex, type 1 diabetes mellitus, autoimmune diseases, immunodeficiency, and chromosomal anomalies.

**Results:**

The Cox model included 493,382 children. VSD was not independently associated with an increased risk of CD (HR 1.25, 97.5% CI 0.85–1.82). In contrast, established comorbidities showed strong associations with CD: type 1 diabetes mellitus (HR 10.26, 97.5% CI 8.15–12.91), chromosomal anomalies (HR 5.20, 97.5% CI 3.67–7.37), and autoimmune diseases (HR 2.07, 97.5% CI 1.39–3.10).

**Conclusion:**

In this large real-world data, isolated VSD was not an independent risk factor for CD, whereas type 1 diabetes mellitus, chromosomal anomalies, and other autoimmune diseases were strongly associated with CD. These findings do not support routine CD screening based solely on the presence of VSD in children.

## Introduction

Celiac disease (CD) is a chronic, immune-mediated enteropathy triggered by gluten ingestion in genetically susceptible individuals. It affects approximately 1% of the population and imposes a particularly high burden in childhood. In pediatric patients, clinical presentation ranges from classic malabsorptive symptoms and failure to thrive to extra-intestinal or even silent forms, making timely recognition challenging (1, 2). Disease development is strongly linked to HLA-DQ2/DQ8 and other genetic and environmental factors, and CD frequently clusters with autoimmune disorders (such as type 1 diabetes mellitus and autoimmune thyroid disease), chromosomal syndromes, and primary immunodeficiencies (3, 4). Consequently, current guidelines recommend targeted serologic screening for celiac disease in children who belong to these well-defined risk groups (5).

Ventricular septal defect (VSD) is one of the most common congenital heart defects in children and is characterized by an abnormal communication between the right and left ventricles (6, 7). Clinical severity ranges from small, hemodynamically insignificant defects that may close spontaneously to large defects that can result in heart failure, pulmonary hypertension, arrhythmias, and aortic regurgitation (7). VSD has a multifactorial etiology and often occurs in association with chromosomal or syndromic conditions, such as Down syndrome, Turner syndrome, and Williams syndrome; these conditions are themselves associated with a higher prevalence of CD (6).

Several observations suggest that an association between VSD and CD is biologically and clinically plausible. CD has been reported to occur more commonly in children with chromosomal anomalies and in those with congenital heart disease compared with the general population (5, 8, 9). CD shares genetic and immunologic pathways with autoimmune and cardiovascular diseases: it is strongly associated with type 1 diabetes mellitus, autoimmune thyroid disease, and systemic lupus erythematosus. In addition, genome-wide studies have identified overlapping susceptibility loci between immune-mediated and cardiovascular conditions (10–12). Congenital heart defects, including VSD, have been linked to immune dysregulation, altered acute-phase protein profiles, and genes involved in both cardiac morphogenesis and immune regulation, such as GATA4 (13–15). Furthermore, CD has been associated with coronary heart disease and cerebrovascular disease (16, 17). Together, these data suggest that children with VSD could be at increased risk for CD through shared chromosomal, genetic, and immunologic mechanisms.

Despite this theoretical overlap, the epidemiologic relationship between VSD and CD remains poorly defined. Existing studies involving pediatric populations with congenital heart disease have been small and have yielded inconsistent results, and no large population-based cohort has specifically examined whether isolated VSD is independently associated with CD after adjustment for established risk factors (8, 9, 18). Clarifying this association is clinically important, as it would inform whether VSD should be considered an indication for targeted screening for CD in childhood. Therefore, using data from a large health maintenance organization, we aimed to evaluate whether children with VSD have an increased risk of developing CD compared with age- and sex-matched controls. As secondary aims, we assessed the associations between CD and its recognized comorbidities, including type 1 diabetes mellitus, other autoimmune diseases, primary immunodeficiency, and chromosomal anomalies, to contextualize the findings related to VSD within the broader risk profile of CD.

## Methods

### Study design and patient population

We conducted a retrospective cohort study using data from Clalit Health Services (CHS), Israel's largest not-for-profit health maintenance organization, which provides comprehensive electronic medical records with extensive coverage and high data completeness across primary, secondary, and tertiary care settings (19–21). The study protocol was approved by the institutional review board of Kaplan Medical Center, and data extraction and de-identification were performed using the Wiser platform (Rehovot, version Alpha) (19–21).

The source population comprised all children insured by CHS who were born in Israel during the study period and had at least 1 year of continuous CHS membership. Children born outside Israel were excluded because their perinatal and early childhood medical records might not have been fully captured in the CHS database, which could lead to incomplete ascertainment of VSD, CD, and relevant comorbidities, potentially introducing bias into follow-up assessment.

Children with VSD were identified using ICD diagnostic codes in the cardiology clinic and hospitalization records. Within CHS, VSD diagnoses are assigned by pediatric cardiologists based on standardized echocardiography reports, and congenital heart defect codes are routinely audited as part of internal quality-assurance procedures, supporting the high validity for VSD classification (19–21). For each child with VSD, we randomly selected a control group of children without any documented VSD or other major structural heart disease, using a 1:10 matching ratio by sex and exact year of birth. Matching by age and sex ensured comparable baseline risk profiles and follow-up duration between the groups.

Comorbid conditions were defined using diagnostic codes from outpatient visits, hospitalizations, and specialty clinics. We considered type 1 diabetes mellitus, autoimmune diseases (including autoimmune thyroid disease, inflammatory bowel disease, rheumatoid arthritis, systemic lupus erythematosus, psoriasis, and psoriatic arthritis), primary immunodeficiency, and chromosomal anomalies as potential risk factors for CD. These conditions are typically chronic and stable and are most often diagnosed early in life. Accordingly, a child was classified as having a given comorbidity if the diagnosis appeared at any time in the CHS record (before or during follow-up). This approach maximizes sensitivity for identifying established CD risk groups while reflecting the long-term nature of these diagnoses.

### IgG-based Cox regression cohort (laboratory-anchored cohort)

**Figure 1:**
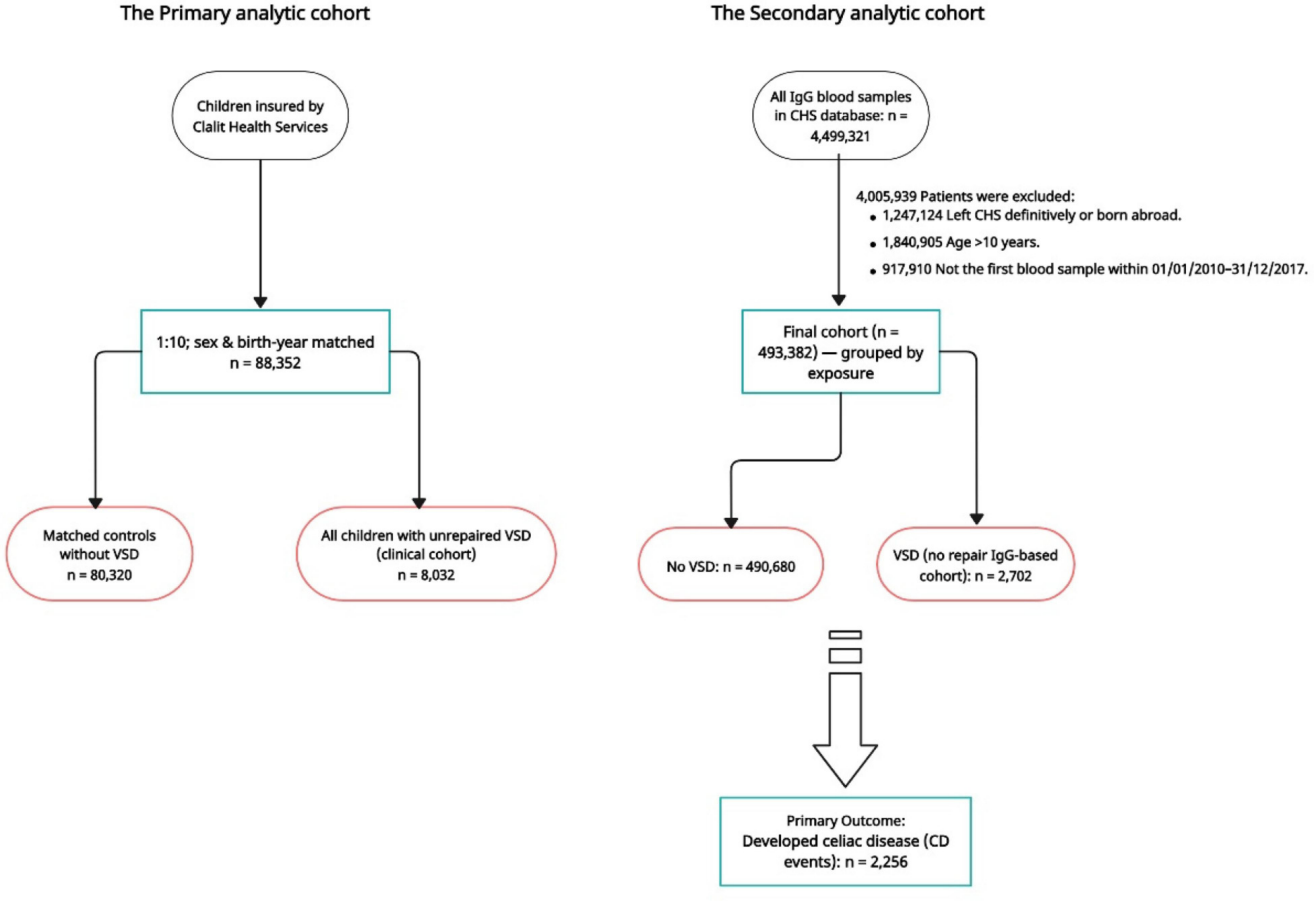
Study flow diagram and analytic cohorts. The figure illustrates the construction of the two analytic cohorts used in the study, both derived from the same underlying Clalit Health Services (CHS) source population. The primary analytic cohort (left) comprises a 1:10 sex- and birth-year-matched clinical cohort, including all children with unrepaired ventricular septal defect (VSD) and matched controls without VSD. This cohort was used for Kaplan–Meier analyses. The secondary analytic cohort (right) is a laboratory-anchored cohort constructed using the first recorded serum IgG test as a methodological anchor to define a uniform index date for follow-up. This cohort was used for multivariable and piecewise Cox regression analyses. IgG was not treated as a biological exposure and was not hypothesized to have a causal role in celiac disease; rather, it was used solely to standardize cohort entry, age eligibility, and calendar time. In this cohort, follow-up began on the date of the first IgG test. The two cohorts partially overlap but are not nested. The numbers shown represent the remaining eligible children after each exclusion step. CHS, Clalit Health Services; VSD, ventricular septal defect; CD, celiac disease; IgG, immunoglobulin G.

To construct the Cox regression cohort, we used the CHS central laboratory database to identify all children aged 0–10 years who had at least one recorded serum IgG test. Cohort entry (index date) was defined as the date of the first available IgG test performed between 1 January 2010 and 31 December 2017. This laboratory-anchored approach was used solely to define a uniform, objectively recorded index date and to apply consistent eligibility criteria for age and calendar period, thereby enabling adjusted time-to-event modeling in a large population-based cohort. IgG was not treated as a biological exposure, and we did not hypothesize any causal role for IgG in the development of celiac disease. Children born outside Israel or those who left CHS definitively were excluded to ensure complete capture of diagnoses and follow-up. Follow-up began on the index IgG date and ended at the earliest occurrence of incident celiac disease diagnosis, 10 years of follow-up, death, loss of CHS membership, or the end of the study period. Children with evidence of celiac disease prior to the index date were excluded to ensure incident outcome ascertainment. VSD status and comorbidities were determined from CHS clinical records (as described above), and children were grouped according to VSD exposure within the IgG-based cohort (Figure 1; Supplementary eFigures S1A–C).

### Statistical analysis

The primary analytic cohort consisted of the matched VSD and control children described above. Baseline characteristics were summarized as means with standard deviations or medians and interquartile ranges for continuous variables and as counts and percentages for categorical variables. Comparisons between VSD and control groups were assessed using the *χ*^2^ test (or Fisher's exact test, when appropriate) for categorical variables and the Student's *t*-test or Wilcoxon rank-sum test for continuous variables.

Time-to-event analyses were used to evaluate the association between VSD and incident CD. Follow-up began at cohort entry (birth or first registration with CHS, whichever occurred later) and ended at the earliest of CD diagnosis, 10 years of follow-up, death, loss of CHS membership, or the administrative end of the study period. Children who did not develop CD were censored at the time of loss to follow-up or death.

We constructed 10-year Kaplan–Meier curves to estimate CD-free survival, stratified by VSD status and sex. Curves for children with and without VSD were compared using two-sided log-rank (*χ*^2^) tests; these comparisons were considered descriptive.

We fitted multivariable Cox proportional hazards models, using time since cohort entry as the time scale, to estimate hazard ratios (HRs) and 97.5% confidence intervals (CIs) for incident CD. We reported 97.5% CIs (two-sided *α* = 0.025) to provide more conservative inference in the context of a very large sample size and multiple covariates, thereby reducing the likelihood of false-positive findings. The main exposure was VSD status. The models were adjusted for sex and the comorbidities defined in Section “Study design and patient population,” including type 1 diabetes, autoimmune diseases, primary immunodeficiency, and chromosomal anomalies. The proportional hazards assumption was assessed using Schoenfeld residuals and by inspection of log–log survival plots. To explore potential time-dependent effects, we also fitted piecewise Cox proportional hazards models for prespecified follow-up intervals (0–4, 4–7, and 7–10 years). Precision in the piecewise Cox models was evaluated based on the width of confidence intervals and whether confidence intervals crossed 1. Hazard ratios that could not be estimated were interpreted as evidence of sparse data or separation due to very small subgroup sizes. All statistical analyses were performed using R statistical software (R Foundation for Statistical Computing, Vienna, Austria).

## Results

The study compared the prevalence of various health conditions between the VSD group and a control group matched by sex and age. Baseline characteristics of the matched VSD and control cohorts included in the survival analyses are summarized in Table 1.

**Table 1:** Baseline characteristics of the sex- and birth-year-matched Kaplan–Meier cohort.

Variable	VSD group (*n* = 8,032), *n* (%)	Control group (*n* = 80,320), *n* (%)	*P*-value^a^
Autoimmune disease	11 (0.14)	44 (0.06)	**<0**.**01**
Diabetes mellitus type 1	12 (0.14)	74 (0.09)	0.12
Chromosomal anomalies	136 (1.7)	110 (0.14)	**<0**.**01**
Primary immunodeficiency	2 (0.02)	6 (0.01)	0.12

Bold values indicate statistically significant results at α = 0.025 (97.5% confidence interval excluding 1).

^a^ *P*-values were calculated using the chi-square test or Fisher's exact test, as appropriate.

Percentages are calculated within each study group.

### Kaplan–Meier curves

The Kaplan–Meier cohort comprising 88,352 children, including 8,032 with VSD and 80,320 matched controls (1:10 ratio).

**Figure 2:**
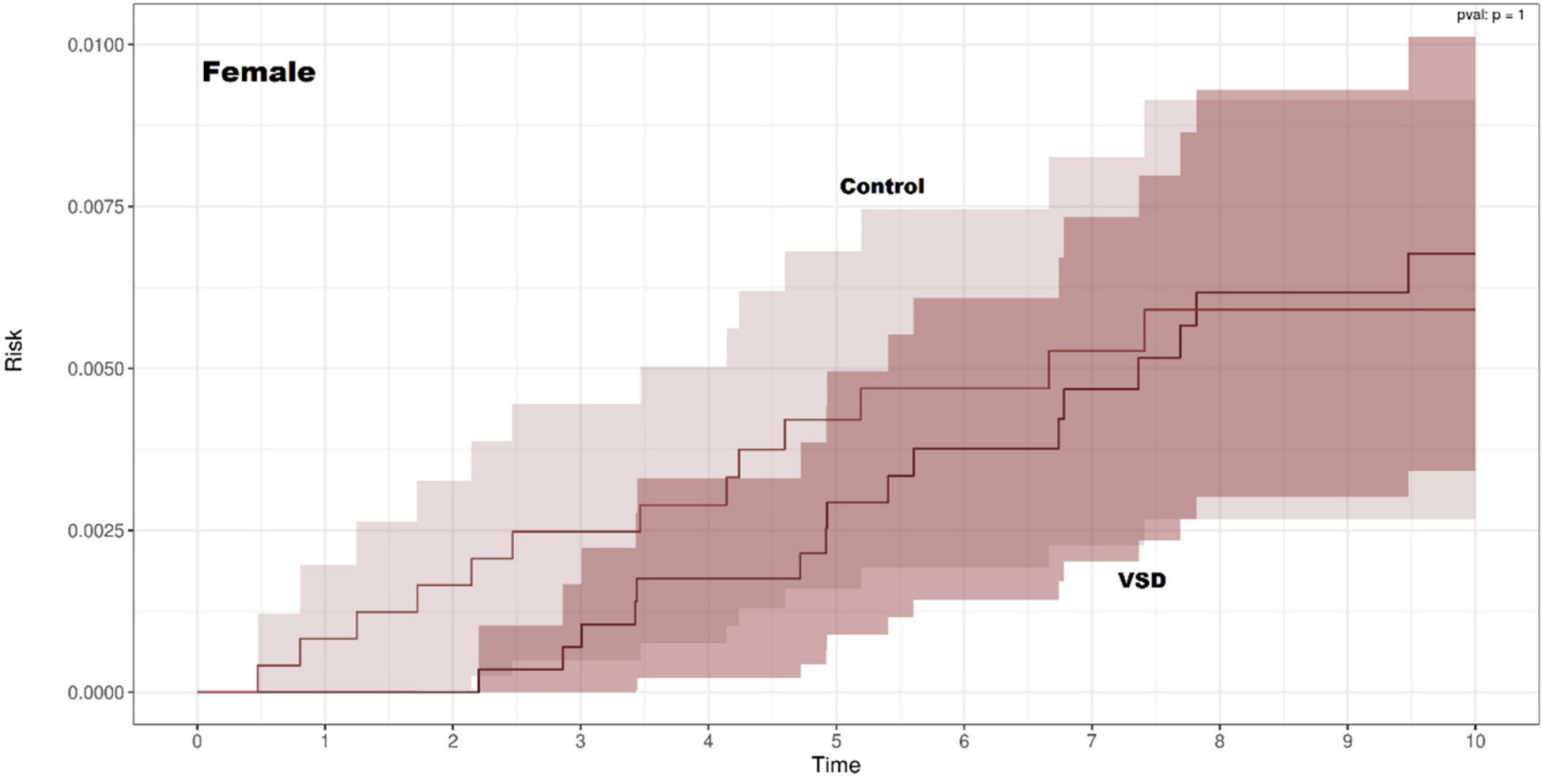
Kaplan–Meier curves of celiac disease-free survival among females with and without ventricular septal defect.

**Figure 3:**
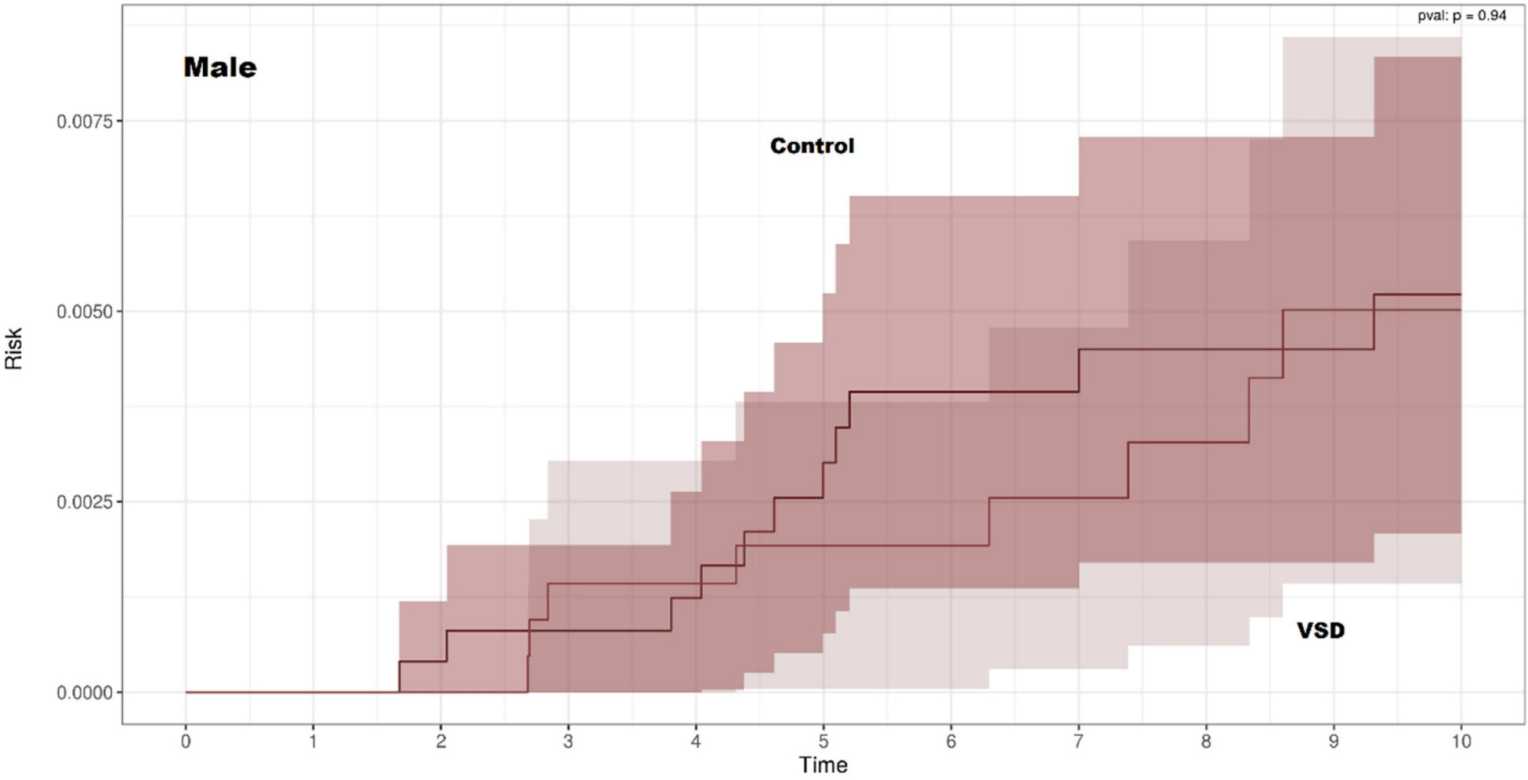
Kaplan–Meier curves of celiac disease-free survival among males with and without ventricular septal defect.

Kaplan–Meier curves for CD-free survival were nearly identical for children with and without VSD in both sexes (Figures 2, 3). There was no significant difference in CD-free survival according to VSD status among females (log-rank *p* = 1.00) or males (*p* = 0.94). Thus, the non-parametric analysis did not show an association between VSD and the subsequent development of CD.

The multivariable Cox proportional hazards model included 493,382 children from CHS (Table 2). After adjusting for sex, age, type 1 diabetes mellitus, autoimmune diseases, primary immunodeficiency, and chromosomal anomalies, VSD was not independently associated with incident celiac disease (adjusted HR 1.25, 97.5% CI 0.85–1.82) (Table 3).

**Table 2:** Baseline characteristics of the Cox regression cohort (*n* = 493,382).

Feature	*N* = 493,382 (%) or mean (±SD)
Autoimmune diseases	2,689 (0.5)
Chromosomal anomalies	821 (0.17)
Type 1 diabetes mellitus	1,259 (0.26)
Ventricular septal defect (VSD)	2,702 (0.54)
Primary immunodeficiency^a^	63 (0.01)
Male sex	252,480 (51.21)
Age (years)	1.25 (± 1.64)

^a^ Primary immunodeficiency was observed in very small number of exposed children.

**Table 3:** Multivariable Cox proportional hazards model for incident celiac disease in the CHS cohort (*n* = 493,382).

Feature	Hazard ratio (IC97.5%)
Primary immunodeficiency^a^	0 (0—inf)
Type 1 diabetes mellitus	**10.26** (**8.15–12.91)**
Chromosomal anomalies	**5.20** (**3.67–7.37)**
Autoimmune diseases	**2.07** (**1.39–3.10)**
Ventricular septal defect (VSD)	1.25 (0.85–1.82)
Age	**0.95** (**0.93–0.96)**
Sex	**0.6** (**0.56–0.64)**

Bold values indicate statistically significant results at α = 0.025 (97.5% confidence interval excluding 1).

^a^ Primary immunodeficiency estimate was non-estimable due to sparse data (very small number of exposed children).

In contrast, several well-established risk factors for celiac disease, consistent with the literature and reflected in current guideline-based screening recommendations, showed strong or moderate associations. Type 1 diabetes mellitus exhibited the highest risk (HR 10.26, 97.5% CI 8.15–12.91), followed by chromosomal anomalies (HR 5.20, 97.5% CI 3.67–7.37). Autoimmune diseases were associated with a moderate increase in risk (HR 2.07, 97.5% CI 1.39–3.10).

Primary immunodeficiency was rare in the Cox regression cohort (63 children; 0.01%; Table 2), and its association with celiac disease could not be reliably estimated in the multivariable model, resulting in a non-estimable HR (0, 97.5% CI 0–∞). This finding reflects limited statistical precision due to sparse data (very small numbers of exposed children), rather than evidence of a null or protective association.

Piecewise Cox models stratified follow-up into 0–4, 4–7, and 7–10 years (Supplementary eTable S1). Hazard ratios for ventricular septal defect remained close to the null across all intervals, with wide confidence intervals crossing 1. In contrast, the elevated risks associated with type 1 diabetes mellitus and chromosomal anomalies persisted throughout all time intervals, with large effect sizes and confidence intervals that did not include 1. For autoimmune diseases, effect estimates were smaller (HRs 1.43–2.12) and less precise, as reflected by wider confidence intervals that crossed 1 in the later follow-up intervals. Primary immunodeficiency was too rare to allow reliable interval-specific estimation.

## Discussion

In this large, real-world pediatric cohort, isolated VSD was not associated with an increased risk of CD. This null association was consistent across Kaplan–Meier analyses and multivariable Cox proportional hazards models.

Importantly, the IgG-based cohort was used only to standardize cohort entry (time zero) for the regression analyses; IgG was not considered a biological exposure, and we neither evaluated IgG levels nor proposed a mechanistic role for IgG in celiac disease. The use of two complementary analytic approaches strengthens the interpretation of our findings. Kaplan–Meier analyses in a sex- and birth-year-matched clinical cohort showed no difference in celiac disease-free survival between children with and without unrepaired VSD. Consistently, multivariable Cox regression in a larger IgG-based cohort demonstrated no independent association between VSD and incident celiac disease after adjustment for established risk factors. The concordant findings across distinct cohorts and analytic strategies support the robustness of our conclusions and suggest that unrepaired VSD alone does not confer an increased long-term risk of celiac disease and, therefore, should not be considered an indication for targeted screening in the absence of other established risk factors. Our findings extend those of previous smaller, clinic-based studies of children with congenital heart disease, which reported a higher prevalence of CD mainly in syndromic or complex cardiac defects; however, these studies did not specifically evaluate isolated VSD or adjust for key comorbidities (8, 9, 18, 22–24).

Several factors may explain this finding. VSD is a common structural defect with heterogeneous severity, and most lesions in our cohort were likely small and hemodynamically insignificant, with minimal long-term systemic impact (6, 7). In addition, the apparent association between congenital heart disease and CD in earlier reports may be attributable to underlying chromosomal or syndromic conditions rather than to VSD itself (8, 9, 18, 22–24). After adjusting for chromosomal anomalies and other major comorbidities, we found no evidence of an independent association between VSD and subsequent CD.

In contrast, we observed the expected associations between CD and established risk factors, including type 1 diabetes mellitus, chromosomal anomalies, and autoimmune diseases, consistent with the well-established literature and current guideline-based recommendations for targeted CD screening in these populations (5–7, 10–12, 25, 26). The presence of these robust associations supports the internal validity of our data. Clinically, our findings do not support adding isolated VSD as an indication for routine CD screening. Screening efforts should remain focused on established high-risk groups, such as those with autoimmune disorders or chromosomal anomalies. In children with isolated VSD, testing for CD should be guided by the presence of suggestive symptoms or additional risk factors.

Although our findings do not support systematic CD screening for isolated VSD, further studies are warranted for other forms of congenital heart disease, particularly complex or syndromic CHD phenotypes in which chromosomal anomalies and immune-mediated comorbidities are more prevalent. Future multicenter studies incorporating detailed cardiac phenotyping (defect type, severity, and repair status) and standardized CD ascertainment could clarify whether any defect-specific risk exists beyond established genetic and autoimmune risk groups.

This study has several limitations that should be considered when interpreting the findings. First, the observational design precludes causal inference, and residual confounding by unmeasured factors (such as family history of CD, HLA genotype, infant feeding practices, and socioeconomic status) cannot be excluded. Second, VSD and CD were identified using routine clinical coding; although VSD diagnoses within CHS are based on standardized echocardiography reports and audited codes, we lacked granular information on VSD anatomy, size, hemodynamic significance, and repair status. Third, although our study used real-world data and included the key variables needed for the analysis (demographics, major diagnoses, and key comorbidities), other potentially relevant clinical variables such as detailed CD serology, histologic Marsh grading, and complete symptom profiles could not be retrieved or adjusted for, as they are not consistently captured within the Weizmann Institute of Science Wiser system. Some covariates (notably primary immunodeficiency) were rare, leading to sparse events and potential separation in some models, with wide or non-estimable confidence intervals. This limits inference for these rare covariates; however, it does not materially affect the primary conclusion, as the VSD estimates remained consistently close to 1 across all time intervals. In addition, the Cox regression analyses were conducted within a laboratory-anchored (IgG-based) cohort, in which cohort entry was defined by the date of the first recorded serum IgG test. This design choice may introduce selection or ascertainment differences, as children undergoing laboratory testing may differ from the general pediatric population in health care utilization or clinical context. However, IgG was used solely as a methodological anchor to define a uniform index date and calendar-time eligibility and was not treated as a biological exposure. Importantly, this criterion was applied uniformly to both the VSD and non-VSD groups in the Cox analyses, and the primary findings were fully consistent with those of the Kaplan–Meier analyses performed in a matched clinical cohort that did not rely on the IgG-based inclusion criterion. Finally, as the study was conducted within a single healthcare organization in Israel, patterns of VSD management, CD diagnosis, and comorbidity profiles may differ in other settings, which may limit the generalizability of our findings. In addition, because this cohort originates from a single geographic setting, regional differences in genetic background, gluten exposure, healthcare access, and diagnostic practices may influence the incidence and ascertainment of CD; therefore, validation of our findings in other countries and ethnically diverse populations is needed.

## Conclusions

In this large, real-world pediatric cohort, isolated VSD was not associated with an increased risk of CD in either Kaplan–Meier or multivariable Cox analyses. Our findings do not support considering VSD alone as an indication for systematic CD screening in children. In contrast, the risk of CD was markedly higher among children with type 1 diabetes mellitus, chromosomal anomalies, and autoimmune diseases. Screening strategies and clinical vigilance should therefore continue to focus on these well-established high-risk groups, while children with isolated VSD should receive standard pediatric follow-up, with testing for CD reserved for those who develop symptoms or have additional risk factors. Future studies should evaluate other congenital heart defect phenotypes and seek to confirm these findings in multicenter, multiethnic cohorts.

## Data Availability

The raw data supporting the conclusions of this article will be made available by the authors upon reasonable request.
