# Supplementary material for: Isolated ventricular septal defect is not a risk factor for celiac disease: evidence from a large real-world data cohort of 493,382 children
Source: Front Pediatr. 2026 Mar 18;14:1751030. doi: 10.3389/fped.2026.1751030 (PMC13038903; doi:10.3389/fped.2026.1751030)
Supplement: Supplementary file 1 [file Supplementaryfile1.docx]

**Supplemnt 1**

**eFigure 1.A Chart Flow: Chart Flow to build VSD group**

**eFigure 1.B Chart Flow: Chart Flow to build Celiac group**

**eFigure 1. C Chart Flow: Chart Flow to build Cox regression group**

**eTable 1. Piecewise Cox proportional hazards**

**eFigure 1.A Chart Flow:** Chart Flow to build VSD group

Chart Flow : VSD group

**ICD-9 :**

745.4

**All VSD**

(n = 16180)

**Diagnosis**

**ICD- 9 :**

Z35.9*/Z35.72*/Z35.7/Z35.7*/Z35.62*/Z35.6/Z35.6*/Z35.55*/Z35.53*/Z35.5/Z35.5*/Z35*/745.5*/745.3*/745.2*/747.0*/746.02*/429.71

**Excluded repaired, provoked, or syndromic VSD** (Remaining eligible VSD: n = 10,747)

**Exclusion**

**Code :**

Left CHS definitively

Born in another country

**Excluded: left CHS definitively or born abroad** (Remaining eligible VSD: n = 8,032)

**Final eligible unrepaired VSD cohort**

8032 patients

**Included**

**eFigure 1.B Chart Flow:** Chart Flow to build Celiac group

Chart Flow : Celiac group

**ICD-9 :**

579.0

**All Celiac**

(n = 29057)

**Diagnosis**

**ICD- 9 :**

Celiac screen positive, Transglutaminase screen positive, transglutaminase IgA > 4.0 (U/mL), Gliadin IgA ab >15(U/mL), Gliadin IgG ab >20 (U/mL)

**Positive laboratory test**

(Remaining eligible n = 8869)

**Exclusion**

**Code :**

Left CHS definitively

Born in another country

**patients who left CHS and patients born in another country** (Remaining eligible n = 7792)

7792 patients

**Included**

**eFigure 1. C Chart Flow :** Chart Flow to build Cox regression group

Chart Flow : Cox regression

**Number of patients included in the final cox regression with VSD : 2702**

**Number of patients included in the final cox regression with CD : 2256**

**All IgG blood sample**

(n = 4,499,321)

**Diagnosis**

**Exclusion if quit CHS or born in another country**

(n = Remaining eligible 3,252,197)

**Only patients aged from 0 to 10 years**

(n = Remaining eligible 1,411,292)

**Exclusion**

**Only first blood sample between 1/1/2010 and 31/12/2017**

Remaining eligible 493382 patients.

**Final eligible unrepaired VSD cohort**

**493382 patients**

**Included**

**eTable 1. Piecewise Cox proportional hazards**

| Variable | 0–4 Years HR (97.5% CI) | 4–7 Years HR (97.5% CI) | 7–10 Years HR (97.5% CI) |
| --- | --- | --- | --- |
| Age (per year) | **1.05 (1.02–1.09)** | **0.86 (0.81–0.92)** | **0.86 (0.79–0.93)** |
| Female sex | **0.59 (0.51–0.68)** | **0.61 (0.52–0.70)** | **0.56 (0.46–0.67)** |
| VSD (no repair) | 1.53 (0.73–3.23) | 1.33 (0.60–2.97) | 1.34 (0.50–3.59) |
| Type 1 diabetes | **8.49 (5.31–13.57)** | **11.12 (7.13–17.35)** | **15.55 (9.93–24.35)** |
| Chromosomal anomaly | **5.72 (2.84–11.50)** | **3.62 (1.50–8.73)** | **4.45 (1.66–11.94)** |
| Autoimmune disease | **2.12 (1.14–3.96)** | 1.43 (0.64–3.19) | 1.64 (0.68–3.97) |

The piecewise Cox regression demonstrates that established risk factors for celiac disease—particularly type 1 diabetes and chromosomal anomalies—exert a strong and persistent influence across childhood, with hazard ratios that increase over time. Type 1 diabetes shows the most pronounced effect, rising from an HR of 8.49 in early childhood (0–4 years) to 15.55 by years 7–10, indicating that the risk of CD in this subgroup not only exists early but intensifies with age. Chromosomal anomalies follow a similar but less steep trajectory, maintaining a two- to four-fold elevated risk throughout follow-up. In contrast, ventricular septal defect without surgical repair does not exhibit a statistically significant association with CD in any time interval. The wide confidence intervals and hazard ratios near 1.0 across all periods indicate no meaningful contribution to long-term CD risk, reinforcing that VSD alone should not be considered an indication for targeted screening. The influence of sex and age shifts over time in expected directions: female sex consistently confers lower risk, and age transitions from a modest positive association in early childhood to a protective effect thereafter, consistent with the typical timing of CD diagnosis. Autoimmune diseases other than type 1 diabetes shows a weaker and less consistent association, likely reflecting heterogeneity within this category or reduced statistical power.

**Kaplan-Meir Curve of Diabetes, chromosomal anomaly and auto immune disease censoring for Celiac (10 years)**

**
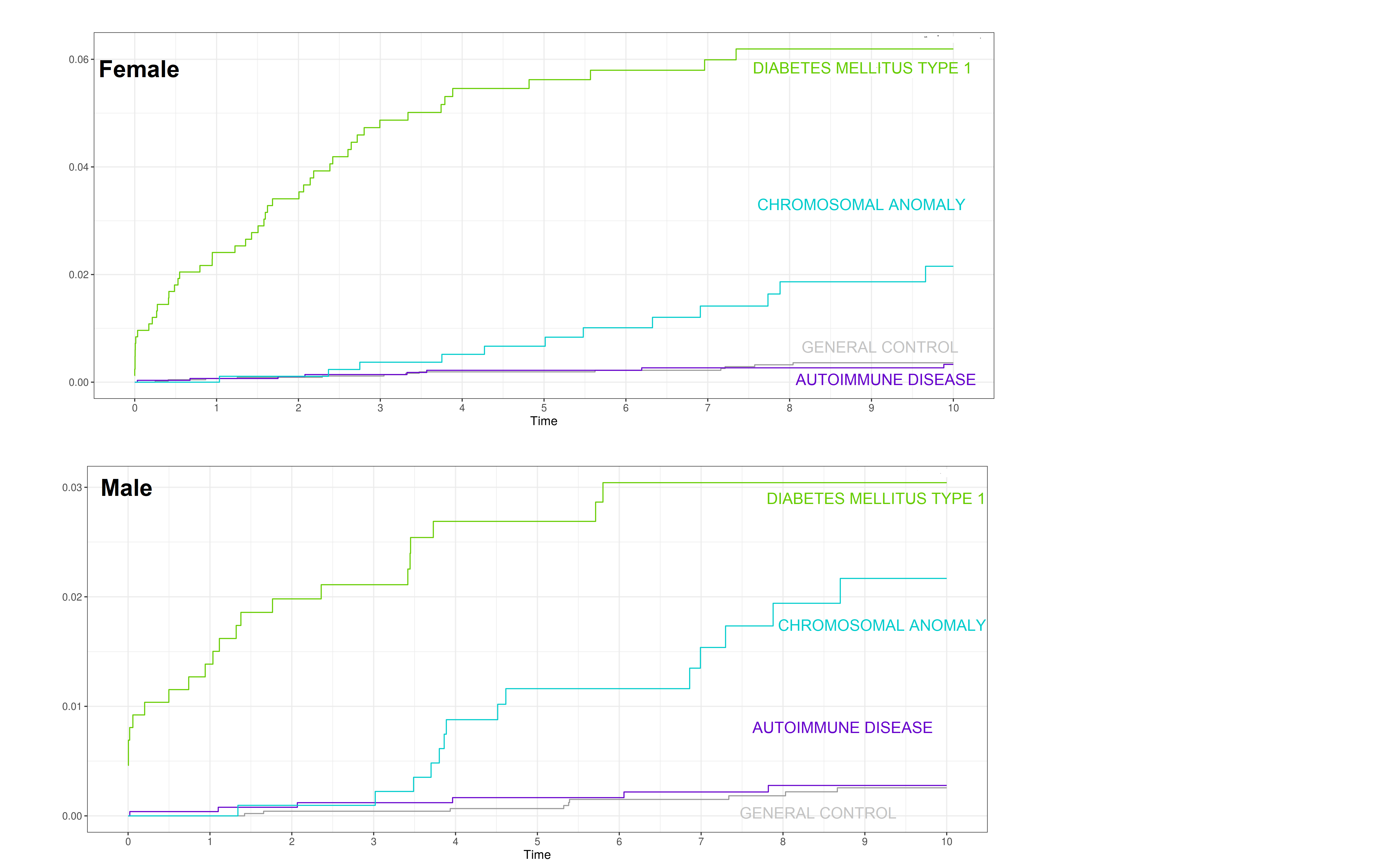
**

**List of ICD-9 used for the covariates**

**chromosomal anomalies**

758.3/758.8/758/758.39/758.0

**Diabetes mellitus type 1**

250.01/250 .03

**Immuno-deficiency**

279.01/279.06

**Autoimmunity**

576.1/571.42/370.33/255.41/ssa or ssb – positive/244.8/242/714/695.6/696.0/696.1
